# Polymorphisms of *XRCC3* and *XRCC7* and Colorectal Cancer Risk in Khorasan Razavi Province, Iran

**DOI:** 10.31557/APJCP.2019.20.7.2153

**Published:** 2019

**Authors:** Jamshid Mehrzad, Mahdieh Dayyani, Mohammadreza Erfanian Khorasani

**Affiliations:** 1 *Department of Biochemistry, Neyshabur Medical Science Branch,*; 3 *Department of Biotechnology, Neyshabur Branch, Islamic Azad University, Neyshabur, *; 3 *Department of Radiation Oncology, Reza Radiotherapy Oncology Center, Mashhad, Iran. *

**Keywords:** XRRC3- XRCC7- polymorphism- colorectal cancer

## Abstract

**Background:**

Colorectal cancer (CRC) is highly prevalent cancer, which should be genetically studied among different peoples of the world**. **

**Objective:**

The aim of this study was to evaluate the effect of *XRCC3*T241M, *XRCC3* *A17893G* and, for the first time, *XRCC7* I3434T polymorphisms on CRC risk in Khorasan Razavi Province, Iran.

**Materials and Methods:**

In this case-control study, 180 patients with CRC and 160 sex- and age-matched healthy controls were studied. Genotypes were determined by RFLP-PCR and ARMS-PCR.

**Results:**

The incidence of CRC was observed to be significantly more in a heterozygous *XRCC3* C/T genotype than in the CC genotype (OR 2.210, 95% CI 1.073-4.548, p=0.030). In the case of the *XRCC7* I3434T polymorphism, CRC risk was significantly (4.3 fold) higher in I/T+T/T variant subjects compared to the I/I genotype (OR 4.394, 95% CI 2.721-7.096, p=0.000). Moreover, the *XRCC3* A17893G polymorphism did not correlate with CRC. In addition, there was no significant difference between the distribution of genotypes of the three studied polymorphisms with demographic and clinicopathological features in the CRC patients.

**Conclusion:**

Polymorphisms of *XRRC3* and *XRCC7* genes are involved in CRC and should be considered as a risk factor.

## Introduction

Colorectal cancer (CRC) is a prevalent cancer worldwide, especially in developed countries, and nowadays, it is increasing in developing countries such as Iran (Haggar et al., 2009). Therefore, CRC should be studied in terms of diagnosis, prevention and treatment. 

Human beings are exposed to a large number of exogenous and endogenous agents that damage genes. Harming agents include various types of air pollution, some types of food and diet, Tobacco, Alcohol, ionizing radiation, ROS and several other factors (Basu, 2018). If damages to a gene are not repaired, they will be mutated in the next generation of cells (Bishehsari et al., 2014). A large number of such mutations can lead to tumors and cancers, such as CRC. Depending on the type of damage, there are several pathways for restoration of genes. The main pathways to repair DNA are excision repair (ER) and double-strand break repair (DSBR). DSBR consists of two types of mechanisms, including homologous recombination (HR) and non-homologous end joining (NHEJ) (Hakem, 2008). In each mechanism, various proteins and enzymes, including *XRCC3* and DNA-dependent protein kinase (DNA-PK), contribute to the repair of damaged DNA (Nissar et al., 2014; Chen et al., 2012). If repair factors themselves have problems, repair will likely be confronted with problems and thus mutations and then cancer may occur.

As we know, after completing the human genome project, it became clear that our genes have a lot of polymorphisms. One of the most abundant types of polymorphisms is single nucleotide polymorphism (SNP). The existence of a SNP in the exon region of a gene may lead to a change in one of the amino acids in the protein. By replacing an amino acid in a protein, the conformation and function of that protein may be altered and it cannot act properly. If SNPs are present in non-exon regions of a gene, they may affect regulation of transcription or mRNA processing and turnover (Hrdlickova et al., 2014). Thus, in general, polymorphisms may play an important role in the development of a cancer. 

X-ray repair complementing defective repair in Chinese hamster cells 3 (*XRCC3*) and X-ray repair complementing defective repair in Chinese hamster cells 7 (*XRCC7*) are two genes that were studied in the present work. The protein encoded by the *XRCC3* gene is *XRCC3* and the product of the *XRCC7* gene is DNA-PKcs. Both of the proteins are involved in the DSBR mechanism, i.e. *XRCC3* in HR and DNA-PK in NHEJ (Nissar et al., 2014, Chen et al., 2012). A common SNP in exon 7 of the *XRCC3* gene leads to replacement of an amino acid at position 241 (Thr241Met) of the protein and the IVS5–14 (A17893G, rs1799796) polymorphism is in intron 5 of the gene (Mandal et al., 2010). So far, several studies have been conducted on these SNPs in various cancers and in different human races. Some of these studies have proven the association between these polymorphisms and cancer while some others have found no relationship between them, such as studies on colorectal adenoma (Tranah et al., 2004), lung (Ryk et al., 2006) and breast cancer (Mohammed-Ali et al., 2016; SU et al., 2015). Further, there are scarce studies on the association between the common genetic Ile3434Thr polymorphism (rs7830743) *XRCC7* and cancers. Moreover, few studies conducted on *XRCC3* have also reported controversial results, with some studies confirming the relationship between *XRCC3* and cancer and some others rejecting it (Zhang et al., 2013; Rahimi et al., 2012).

There are different ethnic groups such as Persians, Turks, Kurds, Baluchs, Arabs and others in different cities and provinces of Iran. Khorasan Razavi Province, northeastern Iran, has heterogeneous population. Thus, it may be genetically diverse and different from other parts of the country. Therefore, with regard to what mentioned above, for the first time, we investigated the effect of two repair gene polymorphisms on CRC risk among people in northeastern Iran.

## Materials and Methods

*Study Participants and Blood Collection*

The present project was approved by the Ethics Committee of the Islamic Azad University (approval number: IR.IAU.NEYSHABUR.REC.1395.9). In this case-control study, approximately 5 ml of peripheral whole blood were collected from each of the 180 cases of sporadic CRC and 160 healthy individuals, as controls, in EDTA containing tubes and stored at -80°C until analyzed. The mean (±SD) age of the patients was 57.9±14.4 years and they included 77 (42.8%) females and 103 (57.2%) males. The control group, including 70 (43.8%) females and 90 (56.2%) males, had an average (±SD) age of 57.2±13.9 years. The patients were randomly selected from CRC patients referred to the Reza Radiotherapy and Oncology Center in Mashhad and the 22 Bahman Hospital in Neyshabur. Healthy individuals were selected from among volunteers whose age and gender were matched to the patients. Before collecting blood samples and filling out questionnaires, information was provided to the patients and the healthy individuals and then written consent forms were obtained from all the participants. The clinicopathological information of the patients was collected by the manual review of their pathology reports and hospital records.

*DNA Extraction and Genotyping*

Kits required to isolate genomic DNA were purchased from the Korean company, Bioneer. Thus, DNA extraction was carried out according to the manufacturer’s instructions and stored at –20˚C until used for genotyping. Genotyping of two SNPs was carried out by restriction fragment length polymorphism-PCR (RFLP-PCR) in the *XRCC3* gene and by an amplification refractory mutation system-PCR called the ARMS-PCR method in the *XRCC7* polymorphism, using forward (F) and reverse (R) primers listed in Table 1.

**Figure 1:**
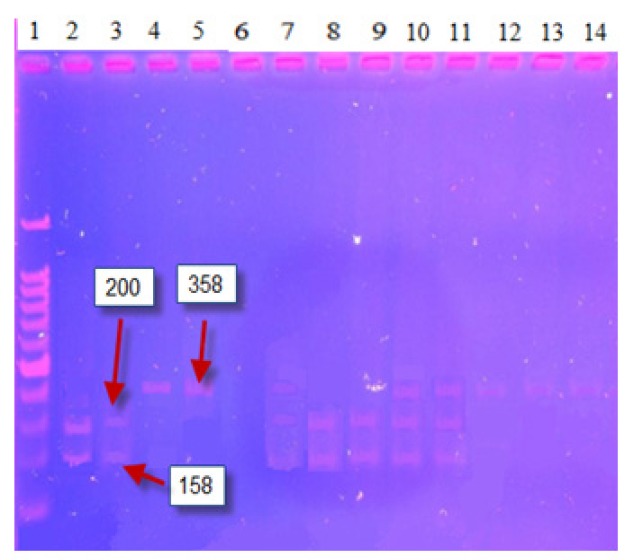
*XRCC3* (rs861539 C>T) Digested PCR Products of Thirteen Samples. Lane (1) 100 bp ladder, Sample Lanes (7, 10 and 11) are CT Genotype, Sample Lanes (4, 5 and 12-14) are CC Genotype. Lanes (2, 3, 8 and 9) are TT

**Figure 2:**
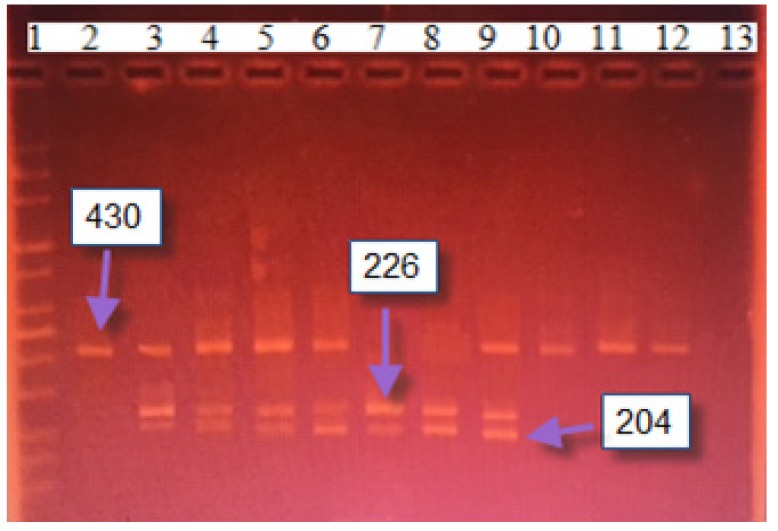
*XRCC3* (rs1799796 G>A) Digested PCR Products of Twelve Samples. Lane (1) 100 bp ladder, Sample Lanes (2, 6-9) are CT Genotype, Sample Lanes (4,7-9) are CC Genotype. Lanes (12-14) are TT

For PCR-RFLP in 25 μl reaction, the following materials were used: 250 μm dNTPs, 1.5 mM MgCl_2_, 100 ng DNA, 12.5 pmol of each primer, and 1 U Taq DNA polymerase. The 358bp amplified product for *XRCC3* (*rs861539*) was digested with FatI. The wild-type allele Thr was identified by the presence of a 358bp band (indicative of the absence of the FatI cutting site), while the mutant allele Met was detected by 200 and 158bp bands (Figure 1). For *XRCC3* (rs1799796), a 430bp amplified product was digested with AluI. The wild-type allele G was identified by the presence of a 430bp band (indicative of the absence of the AluI cutting site), while the mutant allele A was detected with the appearance of 226bp and 204bp bands (Figure 2) (Su et al., 2015).

**Figure 3:**
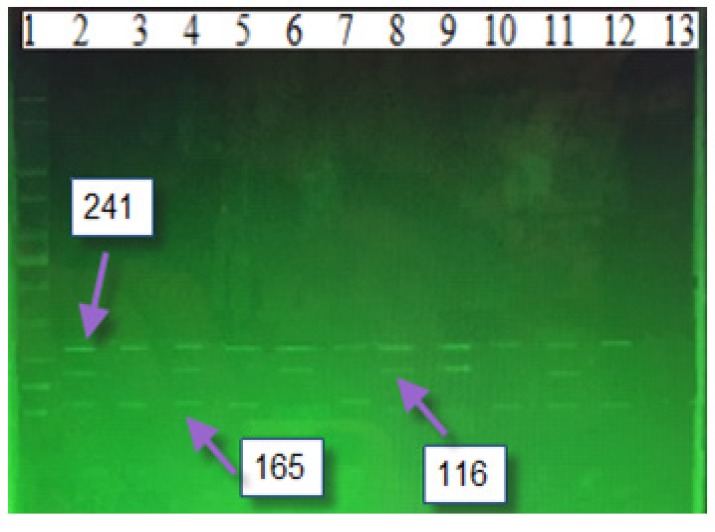
XRCC7 (rs7830743) A 241-bp DNA Segment was Amplified Using Common Forward (CF) and Common Reverse (CR) Primers, while 116bp and 165bp Allele-Specific Amplicons were Amplified Using CF-SC and CR-ST Primer Pairs, Respectively. Lane (1) 100 bp Ladder, Sample Lanes (3, 8 and 13) Showed CG Genotype. While Sample Lanes (4, 6, 11-12) Show the TT Genotype. Sample Lanes (5, 7, 10, 14-16) showed genotype TC. Sample Lane (2) Show Control TT Genotype

In performing ARMS-PCR, each 25 μl reaction tube contained 0.1U Taq DNA polymerase, 250 μm dNTPs, PCR 10X buffer, 3.0 mM MgCl_2_, 10 pmol common primers, 24 pmol C or T primers, and 10-15 ng extracted DNA. A 241-bp DNA segment was amplified using common forward (CF) and common reverse (CR) primers, while 116bp and 165bp allele-specific amplicons were amplified using CF-SC and CR-ST primer pairs, respectively (Figure 3). For each sample, two PCR reactions with three primers were performed: CF and CR were common in the both reactions, while SC and ST were specific primers for each reaction (designated as “C” and “T” reactions, respectively) (Rahimi et al., 2012).

*Statistical Analysis*

The Hardy-Weinberg equilibrium was tested to compare the frequencies of the observed genotype with the estimated values within the control group using the chi-square test, which is available at the http://www.oege.org/software/hwe-mr-calc.shtml (Rodriguez et al., 2009). Then, the related p-value was calculated, which is available at the https://www.socscistatistics.com/pvalues/chidistribution.aspx. Moreover, the genotype and allele frequencies were compared in different groups using the chi-square test and the Fisher’s exact test (two-tailed). The odds ratios (OR) and 95% confidence intervals (CI) were calculated by means of a logistic regression model. SPSS 20.0 (SPSS Inc, Chicago, Illinois) was used to perform statistical calculations. All p-values less than 0.05 were considered significant.

## Results

After doing laboratory work, collecting demographic and clinical data and performing statistical analysis, our findings are summarized in Tables 2 and 3.

Regarding the p-values in Table 2, which were all more than 0.05, there was no significant difference between the distribution of the genotypes of the three studied polymorphisms with gender, age, tumor site, stage, grade and tumor size in the CRC patients.

According to other results of the present study, which are presented in Table 3, some genotypes of *XRCC3* Thr241Met and *XRCC7* Ile3434Thr were involved in the development of CRC, but the *XRCC3* rs1799796 polymorphism did not correlate with this cancer. Accordingly, the incidence of CRC was significantly more in the heterozygous *XRCC3* 241Thr/Met (CT) genotype than in the CC genotype (OR 2.210, 95% CI 1.073-4.548 and p-value 0.030). Generally, individuals with the T allele are more likely to have CRC (OR 2.213, 95% CI 1.430-3.425 and p-value 0.000). In the case of the *XRCC7* Ile3434Thr polymorphism, CRC risk was significantly (4.3 fold) higher in AG+GG variant subjects compared to the AA genotype (OR 4.394, 95% CI 2.721-7.096 and p-value 0.000). Therefore, the CT marker is co-dominant and the C allele is not dominant or recessive. In general, those who simultaneously had T and C alleles were more likely to have colorectal cancer (OR 2.213, %95 CI 1.430-3.425 and p-value 0.000). In the case of the Ile3434Thr *XRCC7* polymorphism, the risk of CRC in individuals with a heterozygote AG (Ile/Thr) genotype was 2.2 times higher than those with normal AA homozygote, although the p-value was greater than 0.05. Also, the risk of CRC was significantly 4.3 fold greater in AG+GG variant subjects compared to the AA genotype (OR 4.394, %95 CI 2.721-7.096 and p-value 0.000). Thus the AG is a co-dominant marker.

**Table 1:** Primers Used to Determine the Polymorphisms in* XRCC3* and *XRCC7* Genes

SNPs		Primers	Annealing temperature
*XRCC3* (rs861539) (C>T)	1F	5’- GACACCTTGTTGGAGTGTGT -3’	55°c
	2R	5’- GTCTTCTCGATGGTTAGGCA -3’	
*XRCC3* (rs1799796) (17893 A > G)	F	5’- GG AACCAGTTGTGTGAGCCT -3’	55°c
	R	5’- CCTGGTTGATGCACAGCACA -3’	
*XRCC7* (rs7830743) (T>C)	3CF	5'-CAAGCCAAAAAGGGAAAGTG-3'	56°c
	4CR	5'-GGCTCAAAGTCTCCTCTGGA-3’	
	4SF	(C allele): 5’-TGCAGTTCT GCAGAATCA G-3’	
	5SR	(T allele): 5’-CTTTGGTGTCCTTGATAGTTA T-3	

1 , forward;

2 , revers;

3 , common forward;

4 , common reverse;

5 , spesific forward;

6 , spesific reverse

**Table 2:** The Association Polymorphisms of DNA Repair Genes with Clinicopathological and Demographic Features in 180 Colorectal Cancer Patients

Factor n=Numbers	*XRCC3 Thr241Met *(*rs861539*) C>T			*XRCC3* (*rs1799796*) A>G			*XRCC7 Ile3434Thr *(*rs7830743*) A>G		
	CC n (%)	CT +TT n(%)	*P*	AA n(%)	AG+GG n(%)	*P*	AA n(%)	AG+GG n(%)	*P* a
Gender			0.22			0.431			0.538
Female (n=77)	35 (45)	41 (53)		48 (62)	25 (32)		47 (61)	29 (38)	
Male (n=103)	37 (36)	62 (60)		60 (58)	41 (40)		57 (55)	44 (43)	
Age			0.262			0.845			0.558
<50 (n=49)	16 (32)	33 (67)		31 (63)	17 (35)		25 (51)	23 (47)	
50-65 (n=63)	25 (40)	37 (59)		36 (57)	25 (40)		39 (62)	24 (38)	
>65 (n=60)	28 (47)	30 (50)		36 (60)	21 (35)		35 (58)	23 (38)	
Tumor site			0.303			0.729			0.73
Colon (n=48)	23 (48)	25 (52)		28 (48)	19 (40)		29 (60)	21 (43)	
Rectum (n=130)	49 (38)	78 (60)		78 (60)	47 (36)		75 (58)	52 (40)	
Stage			0.363			0.521			0.788
I (n=1)	0 (0)	1 (100)		1 (100)	0 (0)		1 (100)	0 (0)	
II (n=27)	8 (30)	19 (70)		14 (52)	11 (40)		14 (52)	12 (44)	
III (n=36)	17 (47)	19 (53)		23 (64)	12 (33)		22 (61)	14 (39)	
IV (n=27)	13 (48)	14 (52)		14 (52)	11 (41)		18 (67)	9 (33)	
Grade			0.396			0.84			0.645
bWD (n=65)	26 (46)	38 (58)		42 (65)	23 (35)		36 (55)	29 (45)	
cMD (n=70)	28 (40)	41 (58)		39 (58)	28 (40)		39 (58)	29 (41)	
dPD (n=5)	3 (60)	2 (40)		3 (60)	2 (40)		3 (60)	2 (40)	
eUD (n=2)	2 (100)	0 (0)		1 (50)	0 (0)		2 (100)	0 (0)	
Tumor Size (cm)			0.644			0.073			0.837
<5 (n=53)	17 (32)	36 (68)		40 (75)	12 (23)		30 (57)	22 (42)	
5-10 (n=30)	12 (40)	17 (57)		16 (53)	14 (47)		15 (50)	14 (49)	
>10 (n=6)	3 (50)	3 (50)		4 (67)	2 (33)		3 (50)	3 (50)	
Local tumor invasion			0.767			1			1
T1-T2 (n=14)	5 (36)	9 (64)		9 (64)	5 (35)		8 (57)	6 (43)	
T3-T4 (n=59)	25 (42)	34 (58)		35 (59)	21 (36)		34 (58)	24 (40)	
Lymph nodes involvement			0.223			0.42			0.612
N0 (n=30)	10 (33)	20 (67)		16 (53)	13 (43)		17 (57)	12 (40)	
N1-N2 (n=41)	21 (51)	20 (49)		28 (68)	12 (29)		24 (58)	18 (44)	
Distant metastasis			0.611			1			1
M0 (n=12)	6 (50)	6 (50)		7 (58)	5 (42)		7 (58)	5 (42)	
M1-M2 (n=37)	16 (43)	21 (57)		20 (54)	14 (39)		21 (57)	15 (40)	

a p-value based on χ^2^ test and Fisher’s exact test (two- sided);

b WD, well-differentiated;

c MD, moderately differentiated;

d PD, poorly differentiated;

e UD, undifferentiated

**Table 3:** Distribution and Correlation of Polymorphisms of DNA Repair Genes in Colorectal Cancer Patients and Controls

	Cancer (total = 180)	Control (total = 160)	Colorectal cancer versus control	
	Number (%)	Number (%)	OR (95% CI)	a*P*
*XRCC3Thr241Met *(*rs861539*) C>T				
CC	63 (35)	87 (54.7)		
CT	93 (51.7)	57 (35.8)	2.210 (1.073-4.548)	*0.031
TT	24 (13.3)	15 (9.4)	0.998 (0.484-2.058)	0.995
CT+TT	117 (65)	72 (45.3)	2.213 (1.430-3.425)	*0.000
*XRCC3* (*rs1799796*) A>G				
AA	108 (60)	91 (56.9)		
AG	51 (28.3)	48 (30.0)	1.065 (0.474-2.390)	0.879
GG	15 (8.3)	12 (7.5)	1.176 (0.500-2.767)	0.71
AG+GG	66 (36.6)	42 (37.5)	0.937 (0.599-1.465)	0.775
*XRCC7 Ile3434Thr *(*rs7830743*) A>G				
AA	82 (45.6)	124 (77.5)		
AG	95 (52.8)	32 (20)	2.232 (0.374-13.982)	0.371
GG	3 (1.7)	2 (1.2)	0.516 (0.081-3.161)	0.466
AG+GG	98 (54.4)	34 (21.2)	4.394 (2.721-7.096)	*0.000

ORs, odds ratio; and CIs, confidence interval were estimated from unconditional logistic regressions, controlling for age and gender.

b p-value based on χ^2^ test and Fisher’s exact test (two- sided).

* Statistically significant.

The *XRCC3* Thr241Met, *XRCC3* rs1799796 and *XRCC7* Ile3434Thr genotypes in the control group were in the Hardy-Weinberg equilibrium, as demonstrated by the lack of any significant difference between their observed and expected frequencies (χ^2^=1.53; p=0.216, χ^2^=2.35; p=0.125, χ^2^=0.00; p=1, respectively). Therefore, this suggests that the control subjects may represent the general population. 

## Discussion

There are several processes to repair DNA damages, and in each mechanism, there are a large number of proteins, including *XRCC3* and *XRCC7*. If any of such processes has problems, the repair will not be performed correctly and will appear as a mutation in a new cell.

The present study was conducted to determine whether the *XRCC3*Thr241Met, *XRCC3* *rs1799796* and *XRCC7* Ile3434Thr polymorphisms were different in CRC and healthy individuals as they are susceptibility factors for the development of CRC. 

In humans, the *XRCC3* protein is one of the RAD51 parallogues and participates in HR through interaction with other proteins. There are studies that have shown that *XRCC3* deficiency may interfere with DSBR (Deans et al., 2003; Thacker, 2005). It is therefore assumed that this protein plays an important role in the DNA repair. The presence of the *rs1799796* polymorphism in the intron region of the *XRCC3* gene, if reduces gene expression, may interfere with DNA repair and contribute to the abundance of mutations and cancer risk. However, according to the results of our study, it was realized that this polymorphism did not affect the regulation of *XRCC3* gene expression. Moreover, the findings of the present study revealed that there was no significant difference in the distribution of the frequency of the *XRCC3* *rs1799796* polymorphism between the CRC and control groups (all p-value>0.005). It should be noted that a number of studies have similar results to those of our study. For example, a study found no association between this polymorphism and colorectal adenoma in the United States (Tranah et al., 2004) with breast cancer in Saudi Arabia and Taiwan (Mohammed et al., 2016; SU et al., 2015). However, a significant relationship was observed between the *XRCC3* *rs1799796* polymorphism and ovarian cancer risk in a study conducted in China (Yuan et al., 2014).

With regard to the *XRCC3*Thr241Met polymorphism, several investigations observed that the presence of Met with a hydrophobic methyl sulfur group instead of Thr with a hydrophilic hydroxyl group at the position 241 of the XRCC4 protein was significantly related to high DNA adduct levels (Matullo et al., 2001; Yoshihara et al., 2006). Scientists have determined that *XRCC3* has only a domain with functional activity and the 241Thr/Met variant is located in this domain. Therefore, it is believed that the *XRCC3* (241Met) variant is involved in susceptibility to cancer (Manuguerra et al., 2006). The present study confirmed that the *XRCC3*Thr241Met polymorphism had a significant relationship with CRC risk (p-value<0.005). The increased risk of cancer associated with this polymorphism has also been proven in several other studies on CRC (Zhao et al., 2012; Nissar et al., 2014; Jiang et al., 2010; Slyskova et al., 2012), gastric cancer (Yan et al., 2009; Fang et al., 2011), ovarian cancer (Yuan et al., 2014), endometrial cancer (Smolarz et al., 2018), and prostate cancer (Mandal et al., 2012). However, some studies have found no significant relationship between this polymorphism and cancers, such as breast cancer (Mohammed et al., 2016) and hepatocellular carcinoma (Avadanei et al., 2018).

Another protein involved in the DSB repair is the DNA-PK protein derived from the *XRCC7* gene (Gapud and Sleckman, 2011). There is a polymorphism in the *XRCC7* gene, which results in the replacement of the hydrophobic isoleucine with the hydrophilic threonine at position 3434 in the FRAP-ATM-TRRAP domain of the DNA-PK protein. Therefore, it can be assumed that this substitution may interfere with the functioning of the protein and thus the DNA repair will not be done properly, which ultimately leads to cancer. However, so far, little research has been carried out on the association between the *XRCC7* Ile3434Thr polymorphism and cancers. According to our knowledge, the present study was the first investigation on CRC, and the findings indicated that the 3,434Thr/Ile variant contributed to this cancer (p-value<0.05). Previously, a study carried out in Iran showed that this polymorphism was also related to thyroid cancer (Rahimi et al., 2012). Therefore, the results of these two studies indicated that the *XRCC7* Ile3434Thr polymorphism could be a tumor marker in Iran. However, a study conducted in China showed that the *XRCC7* Ile3434Thr polymorphism did not correlate with cancer (Zhang et al., 2013).

All conflicting relationships mentioned in various studies are expected in different countries and regions, which may be related to ethnic difference, sample size and various other variables.

The interaction between gene polymorphism with demographic factors and pathological symptoms of CRC was investigated in a limited number of studies, including the current study. However, no significant relationship was found between them in almost all of these studies, which is in line with our findings (all p-values>0.05). For example, a study about ornithine decarboxylase gene polymorphism (+316 ODC1 rs2302615) showed that age, gender, stage, colon/rectum site, tumor grade, and histologic subtype were not related to genotypes and alleles, with all p-values>0.05 (Zell et al., 2009). Another study found that the interaction between transforming growth factor-β1 gene promoter -509C/T polymorphism and CRC was only due to the relationship between gender and stage with CC genotype, but not due to other noted variables (Stanilova et al., 2018). The existence of the CC genotype in men has been associated with cancer development and progression.

In general, it can be concluded that the *XRCC3Thr241Met* polymorphism, but not its *rs1799796 *polymorphism, plays a role in the development of CRC. Moreover, the *XRCC7 Ile3434Thr* polymorphism plays a significant role in increasing CRC risk. However, since it has been less studied, it is suggested to further examine its effect on more samples. According to the findings of the present study, the studied polymorphisms in *XRCC3* and *XRCC7* did not cause any progression, metastasis or other parameters in CRC.
